# The Temperature in Influenza

**Published:** 1890-02-08

**Authors:** 


### THE TEMPERATURE IN INFLUENZA.

Dr. Otto Fräntzel contributes to the *Centralblatt fur Klinische Medicin* a valuable paper on this subject, in which he discusses the question of the pyrexia, giving the curves obtained by the comparison of fifty uncomplicated cases, all over fifteen years of age, in which thermometric observations were made every three hours during the day, and every four hours during the night, and in none of which were any antipyretic drugs employed. Unfortunately the observations of the earlier stages were incomplete, many of the patients not having been seen until the disease was at its height. The highest temperature he found was 41° C.=105·8 F., in 11 cases it exceeded 40° C.=104° F., in a few it did not rise beyond 39° C.=102·2 F., and in two 38° C.=100·4° F. In these, however, the acme of the fever had probably passed. The average duration of the febrile period was 3—4 days, rarely 5, and in two cases only was defervescence delayed beyond the week, viz., to the ninth and tenth days respectively. On the other hand, in eight the fever did not last beyond the first day, but he would not have these taken as representing the percentage of such cases.

For that portion of the curve which represents the onset of the fever, he relied on the observations made on nurses and patients already in the hospital suffering from non-febrile diseases who contracted influenza while there. Of these the temperatures had been taken twice daily for some time previously, so that the commencement could be fixed. He thus obtained two typical curves. In one the rise is sudden and rapid, amounting to 2° – 3° C. = 3 6° – 5·4° F. within twelve hours, and the maximum is reached in twenty-four hours or so, without any intervening remission.

In the other the ascent occupies three or four times as long, the temperature rising about 1° C. = 1·8° F. daily, with remissions in the morning to the extent of half a degree C. In an intermediate type of fever, the temperature at first rises rapidly 1·5° C., and then follows the remissions of the second type. The extreme height reached in the first type is very rarely maintained for more than a day or two, though in two it remained continuously over 40° C. = 104° F. for three days. The remittent form is certainly most frequently observed, and especially in the course of defervescence. In one case, however, it fell 3 4° C. = 6° F. within twenty-four hours! In all the temperature sank for a short time somewhat below the normal, in three to 36° C. = 96·8° F., and in one a little lower.

In some cases a sort of consecutive fever was developed; in four, after a complete intermission of four to six days, the temperature rose again to 38° C. = 100·4° F., and in one to 102° F., though only for twelve hours. Once, and once only, did he observe a true relapse, that is to say, a return not merely of the fever, but of all the symptoms of the disease.

